# AdipoR1 enhances the radiation resistance via ESR1/CCNB1IP1/cyclin B1 pathway in hepatocellular carcinoma cells

**DOI:** 10.1186/s10020-025-01065-0

**Published:** 2025-01-23

**Authors:** Yuhan Gan, Linhui Zhu, Yimo Li, Ruoting Ge, Jiahe Tian, Yuxin Chen, Xiang He, Shumei Ma, Xiaodong Liu

**Affiliations:** 1 School of Public Health, Wenzhou Medical University, Wenzhou, 325035 China; 2 South Zhejiang Institute of Radiation Medicine and Nuclear Technology, Wenzhou Medical University, Wenzhou, 325809 China; 3 Key Laboratory of Watershed Science and Health of Zhejiang Province, Wenzhou Medical University, Wenzhou, 325035 China

**Keywords:** HCC, AdipoR1, Radiation resistance, Cell cycle, Cyclin B1, CCNB1IP1

## Abstract

Hepatocellular carcinoma is one of the most common malignant tumors, and radiotherapy plays a pivotal role in its therapeutic regimen. However, radiotherapy resistance is the main cause of therapeutic failure in patients. Our previous study revealed that Adiponectin Receptor 1 (AdipoR1) is involved in regulating radiation resistance in liver cancer patients treated with stereotactic body radiotherapy. To explore the mechanism, we performed high-throughput transcriptome sequencing of hepatocellular carcinoma cells with stable knockdown of AdipoR1. KEGG enrichment analysis indicated that the cell cycle and ubiquitination degradation pathways may be involved in the regulation of radiation resistance by AdipoR1.The knockdown of AdipoR1 can attenuate the radiation-induced G2/M phase arrest through cyclin B1.By the ubiquitination IP assay and a rescue experiment, we confirmed that CCNB1IP1 regulated the ubiquitination and degradation of cyclin B1. Combined with information from transcription factor database and AdipoR1 transcriptome sequencing, these results showed that estrogen receptor 1 (ESR1) may be a transcription factor of CCNB1IP1. We found that AdipoR1 promoted the translocation of ESR1 from the cytoplasm to the nucleus, and ESR1 inhibited the transcription of CCNB1IP1.Therefore, we propose that AdipoR1 regulates the ubiquitination level, cell cycle progression, and radiation resistance of HCC cells through the “AdipoR1 /ESR1/CCNB1IP1/cyclin B1” axis. This study will promote the development of novel targeted radiosensitizing drugs.

**Supplementary Information:**

The online version contains supplementary material available at 10.1186/s10020-025-01065-0.

## Introduction

Hepatocellular carcinoma (HCC) currently remains one of the leading causes of cancer-related death in the world (Foerster et al. 2022; Ferlay et al. 2019). Most patients present with unresectable disease and a poor prognosis (Li et al. 2022a, b; Akinyemiju et al. 2017). The current treatments for hepatocellular carcinoma mainly include chemotherapeutics, antibody therapeutics, adjuvant therapy, targeting peptides and peptide vaccines, radiotherapy, etc (Dutta and Mahato 2017). Radiation therapy (RT) plays an increasingly important role in the treatment of HCC (Zhu et al. 2019). For instance, low-dose radiotherapy can be used to relieve symptoms in the late stage (Ohri et al. 2016). However, radiotherapy still has some limitations, such as side effects and radiation resistance, and the underlying mechanism of radiotherapy for HCC remains unclear. Therefore, a proper understanding of the potential targets or molecular mechanisms of radioresistance in HCC is crucial.

The cell cycle is a ubiquitous, complex, and highly regulated process leading to the replication of genomic DNA (Nurse 2000; Nasmyth 2001). Cell cycle progression is mainly regulated by a series of checkpoint regulators, including CDK1 and cyclin B1, during the G1/S and G2/M phase transitions (Yang et al. 2011; Wang et al. 2014). Cyclin B1 is a key gene regulating G2/M phase progression and can affect tumor growth and metastasis. It has been reported that cyclin B1 overexpression could promote tumorigenesis and function as a putative oncogene in a variety of cancers (Huang et al. 2012; Xia et al. 2021). The ubiquitin-proteasome system is a master regulator of intracellular protein degradation and turnover, and an imbalance in this system can lead to overexpression of oncoproteins or downregulation of tumor suppressors, which in turn leads to tumorigenesis. Studies have shown that targeting substrate proteins for ubiquitination and degradation can regulate cell cycle progression and control tumor cell growth (Hu et al. 2019; Kwan et al. 2020; Esposito et al. 2020; Arceci et al. 2019). In addition to regulating the cell cycle, the ubiquitination-proteasome system can also regulate the radiation sensitivity of cancer cells (Huang et al. 2021; Kim et al. 2018; Jie et al. 2021; Song et al. 2022; Sun et al. 2020). It has been reported that G2/M phase arrest is inversely correlated with radiosensitivity (Dong et al. 2003; Sánchez-Molina et al. 2011). Therefore, targeting the ubiquitination pathway to regulate the cell cycle and thereby modulate radiosensitivity may provide new directions for radiotherapy and radioresistance.

Adiponectin is an adipokine secreted by adipose tissue that is responsible for balancing glucose and lipid metabolism and has anti-apoptotic and anti-inflammatory properties. Adiponectin acts by interacting with AdipoR1 and AdipoR2, and the discovery of adiponectin and its receptors over the past two decades has led to a better understanding of metabolic disorders development (Kadowaki and Yamauchi 2005; Li et al. 2017a, b; Sun et al. 2019). The latest research showed that AdipoRs dual agonist JT003 could improve metabolic dysfunction-associated steatohepatitis (MASH) and related liver fibrosis through the AMPK, PPARα, and PI3K-Akt signaling pathways, so AdipoRs agonists may be a candidate drug for the development of effective treatments for MASH and related fibrosis (Xu et al. 2020). Most previous studies on adiponectin and adiponectin receptors have focused on obesity-related diseases such as diabetes and metabolic syndrome; however, recent studies have implicated that AdipoR1 is also closely related to tumorigenesis such as breast, colorectal, and gastric cancer development (Pfeiler et al. 2010; Tsukada et al. 2011; Byeon et al. 2010). In previous studies, we have shown that AdipoR1 promotes the radiosensitivity of HCC cells by activating the ferroptosis pathway in HCC cells, but here we found that AdipoR1 can also regulate cell radiosensitivity by mediating another pathway that has not been reported, the cell cycle pathway (Pfeiler et al. 2010; Tsukada et al. 2011; Byeon et al. 2010).

Our earlier research has found that AdipoR1 plays a pivotal role in modulating radiosensitivity among liver cancer patients undergoing stereotactic body radiotherapy (SBRT) (Li et al. 2021). In our quest to fathom the underlying mechanism, we conducted high-throughput transcriptome sequencing on hepatocellular carcinoma cells in which AdipoR1 had been stably knocked down. To explore the downstream pathways of AdipoR1 and its impact on liver cancer cells, probing the mechanisms by which AdipoR1 influences the radiosensitivity of liver cancer. It has potential implications for the development of predictive biomarkers and therapeutic targets for patients with HCC.

## Materials and methods

### Reagents and antibodies

The following antibodies were used: anti-GAPDH antibody (60004-1-lg) were purchased from Proteintech (IL, USA); anti-CCNB1IP1 (ab71997) and anti-Histone H3 (ab194684) antibodies were purchased from Abcam (Cambridge, UK); anti-AdipoR1 (sc-518030), anti-GFP (sc-9996) and anti-MYC (sc-40) antibodies were purchased from Santa Cruz Biotechnology (Texas, USA); anti-FLAG antibody (SLCF9337) was purchased from Sigma (St. Louis, USA); anti-ESR1 (4267 S), anti-Ubiquitin (14049 S), anti-cyclin B1 (12231 S) and anti-CDC2 (77055 S) antibodies were purchased from Cell Signaling Technology (CST, United States). Secondary antibodies goat anti rabbit IgG- (H + L) HRP conjugate (cat. no. 170–6515) and goat anti-mouse IgG-(H + L) HRP conjugate (cat. no. 170–6516) were obtained from Bio-Rad Laboratories (Mississauga, Ontario, Canada). CoraLite 594–conjugated goat Anti-Rabbit IgG (H + L) (Cat No. SA00013-4) was purchased from Proteintech (Rose-mont, IL). Cycloheximide(CHX), chloroquine(CQ), carbobenzoxy-Leu-Leucinal (MG132) were purchased from MedChemExpress (MCE, USA).

### Cell lines and cell culture

Human HCC cell lines MHCC-97 H, HepG2, and human embryonic kidney cell 293T were obtained from the Chinese Academy of Sciences cell bank (Beijing, China) and confirmed by short tandem repeat (STR). All cells were routinely cultured in Dulbecco’s modified Eagle’s medium (DMEM) (Gibco, United States) containing 10% fetal bovine serum (Gibco, United States) and 1% penicillin/streptomycin (cat. no. 10378016, Life Technologies) at 37℃ in a humidified atmosphere of 5% CO_2_.

### RNAseq for AdipoR1 knockdown and control cell

Total RNA was extracted from AdipoR1 knockdown and control MHCC-97 H cells using a TRIzol reagent. Samples were transferred to Novogene (Novogene, Beijing) for RNA isolation, quality control, library preparation, and sequencing.

### Irradiation

An X-ray generator (X-RAD 320 ix, Precision X-ray Inc, North Branford, CT, United States) was utilized to deliver radiation at a dose rate of 3.0 Gy/min.

### Lentiviral production

The lentiviral short hairpin RNA (shRNA) vector targeting AdipoR1 (pLKO. 1-shAdipoR1) was constructed according to the Oxidative Medicine and Cellular Longevityprotocol of pLKO. 1-blasticidin vector (Addgene, Cambridge, MA, USA). The forward oligo and reverse oligo were annealed and inserted into the pLKO. 1-blasticidin vector. Lentiviruses were produced in 293T cells after cotransfection of pLKO. 1-shAdipoR1 or pLKO. 1-shScramble, packing plasmid psPAX2, and envelope plasmid pMD2G. The virus-containing supernatant was collected 48 h after transfection, filtered and infected with target cells in the presence of 10 µg/ mL polyene (Sigma-Aldrich, H9268), followed by drug selection with 7 µg/ mL insecticide for one week. AdipoR1 shRNA sequence: the forward oligo: CCGGCGTCTATTGTCATTCAGAGAACTCGAGTTCTCTGAATGACAATAGACGTTTTTG and the reverse oligo: AATTCAAAAACGTCTATTGTCATTCAGAGAACTCGAGTTCT CTGAATGACAATAGACG.

### Plasmid constructions and transfection

Human ESR1、cyclin B1 and CCNB1IP1 were cloned into the indicated vectors, including pcDNA3. 1-FLAG. Human cyclin B1 was cloned into the pcDNA3. 1-MYC. pEGFP-AdipoR1 plasmid was purchased from GenePharma (Shanghai, China). Once the sequencing is complete, the plasmid was extracted by an endotoxin-free plasmid purification kit (Qiagen Inc, Chatsworth, CA) and transfected into cells for verification.

### Transfection of siRNA

When the cells reached approximately 30–50% confluence, they were transfected with siRNA using Lipofectamine 2000 (Invitrogen). For each transfection, 200 nmol of siRNA was added per 100 mm plate (final concentration:40 nM). Serum was added 4–6 h after transfection, and the medium was changed completely on the second day. The expression of this protein was detected by Western blot at 48 h after transfection. The sequences were listed as follows: siRNA for ESR1, CTACAGGCCAAATTCAGATAA.

### Cell cycle analysis

The cell cycle was performed using propidium iodide (PI) staining and analyzed using flow cytometry. The cells were treated with ionizing radiation. After 48 h, cells were collected by trypsinization, washed with PBS, and fixed in 75% ethanol at 4℃ for at least 24 h. Cells were washed twice in PBS, and nuclear DNA was stained with 250 µl propidium iodide (50 µg/ml; SolarBio) in the presence of RNase A (1 µl, 10 mg/ml; SolarBio) in PBS for at least 15 min and then subjected to flowcytometric analysis (ACEA NovoCyte 2040R, USA), collecting 2 × 10^4^ cells each time.

### Western blot

Total protein from MHCC-97 H, HepG2 and HEK-293T were extracted on ice using RIPA Lysis Buffer (Beyotime) supplemented with a protease and phosphatase inhibitor cocktail (Roche, Basel, Switzerland). Extracted proteins were separated by sodium dodecyl (SDS)- PAGE and transferred to a PVDF membrane. After completion of the transfer, membranes were blocked with 5% nonfat milk in TBS/0. 1% Tween 20 for 120 min. Incubation with the primary antibody was conducted overnight at 4℃. Incubation with a peroxidase-conjugated anti-mouse or anti-rabbit secondary antibody (1:10000) was performed for 120 min at room temperature. Finally, chemiluminescent analysis was performed.

### Immunoprecipitation assay

Cells were harvested 48 h after transient transfection and lysed in 1%NP-40 lysis buffer [137mM sodium chloride, 10mM sodium fluoride, 50mM TrIS-HCl (pH7.6), 1mM EDTA, 0.1 mm Na3VO4, 10% glycerol, 1%NP-40 and 1mM PMSF]. Whole cell lysates (WCL) were incubated with the specified antibodies overnight at 4 °C with constant rotation. On the second day, protein G plus -agarose was added and rotated at 4℃ for 2 h. The immune complexes were washed with IP washing buffer (China SGI) for 5 times, separated by SDS-PAGE, and analyzed by Western blot.

### Ubiquitination assays

HA-tagged ubiquitin (UB), FLAG-tagged CCNB1IP1 and MYC-tagged cyclin B1 were simultaneously co-transfected into cells and treated with MG132(10µM) 4 h before cell harvest. The lysates were subjected to immunoprecipitation with FLAG antibodies overnight at 4 °C. Ubiquitinated proteins were determined by immunoblotting with anti-MYC antibodies (Santa Cruz, United States).

### Luciferase reporter assays

Double luciferase–reporter gene determination was conducted to determine whether CCNB1IP1 the downstream target gene of ESR1. The CCNB1IP1 promoter fragments were inserted into the pGL3-Basic vector to generate pGL3-CCNB1IP1. pGL3-CCNB1IP1 promoter was cotransfected into cells with ESR1 overexpression vector or control vector by Lipofectamine 2000. After transfection for 48 h, luciferase activity was determined on a Centro LB 960 Luminometer (Berthold Technologies, Germany) and the activity of renilla luciferase was used as a standardized control.

### Quantitative real-time PCR

Total RNA was extracted from cells with TRIzol solution (TaKaRa, Dalian, China). After the total RNA concentration was determined, the mRNA was reversely transcribed into cDNA using a reverse transcription kit (Takara Bio, Japan). qRT-PCR was carried out on a QuantStudio real-time PCR instrument (Thermo Fisher Scientific, USA) using SYBR Premix Ex Taq II (TaKaRa). The amplification conditions were 95 °C for 30 s, then 40 cycles at 95 °C for 5 s and 60 °C for 34 s. The mRNA levels were normalized to GAPDH. The following primers were used: the relative expression levels of genes were calculated using control GAPDH mRNA and the 2^–ΔΔCt^ method. The following primers were used: GAPDH forward CCATGGGTGGAATCATATTGGA and reverse TCAACGGATTTGGTCGTATTGG; ESR1 forward GCTTACTGACCAACCTGGCAGA and reverse GGATCTCTAGCCAGGCACATTC; AdipoR1 forward CTCATCTACCTCTCCATCGT and reverse GAACACTCCTGCTCTTGTCT; cyclin B1 forward CTGCTGGGTGTAGGTCCTTG and reverse TGCCATGTTGATCTTCGCC; CCNB1IP1 forward CTCGGCCTCCCAAGTAGCT and reverse GTCTTCAGAACCTGATTGGCTAATGAGT.

### Immunofluorescence assays

Cells were seeded on 24-well glass coverslips, the glass coverslips with cells were fixed with 4% paraformaldehyde for 20 min, and then subjected to 0.1% TritonX-100 treatment for 15 min. After blocking the cells with 10% donkey serum for 1 h, the cells were treated with the appropriate primary antibodies overnight. The next day, cells were incubated with the corresponding secondary fluorescent antibodies for 1 h and then incubated with DAPI buffer at 37 °C for 10 min.

### CCK-8 assay and colony formation

Cells were seeded in 96-well plates (2 × 103 cells/well), irradiated with different doses of 0, 2, 4, 6 and 8 Gy for 96 h, and CCK-8 (CCK-8, Toshi Laboratory, Japan) was added to each well. Cells were incubated for 2 h. Absorbance was measured using a microplate reader at a wavelength of 450 nm. The proliferation rate of the cells was calculated by the following formula: cell viability=(OD experimental group − OD blank)/(OD control group − OD blank)× 100%. For colony formation assays, hepatoma cells were plated in 6-well plates and cultured in DMEM (Invitrogen) containing 10% fetal bovine serum (Gibco) at 37 °C and 5% carbon dioxide. After 24 h, cells were irradiated with different doses of 0, 2, 4, 6and 8 Gy, respectively. Cells were cultured for 14 days, then fixed with 4% paraformaldehyde (Solarbio, Beijing, China) for 30 min and stained with crystal violet staining (Solarbio, Beijing, China) for 15 min.

### Statistical analysis

Statistical analyses were performed using GraphPad Prism 8 software (San Diego, CA, USA). Differences between the two groups were evaluated with Student’s t test (two-tailed). A multivariate analysis of variance was performed to assess the data obtained from two or more groups. The survival curve was generated with Kaplan–Meier method. Experimental results are presented mean ± SD. Data was considered as statistically significant when the p value was less than 0.05.

## Results

### AdipoR1 regulates ionizing radiation-induced G2/M phase arrest through cyclin B1

**Fig. 1:**
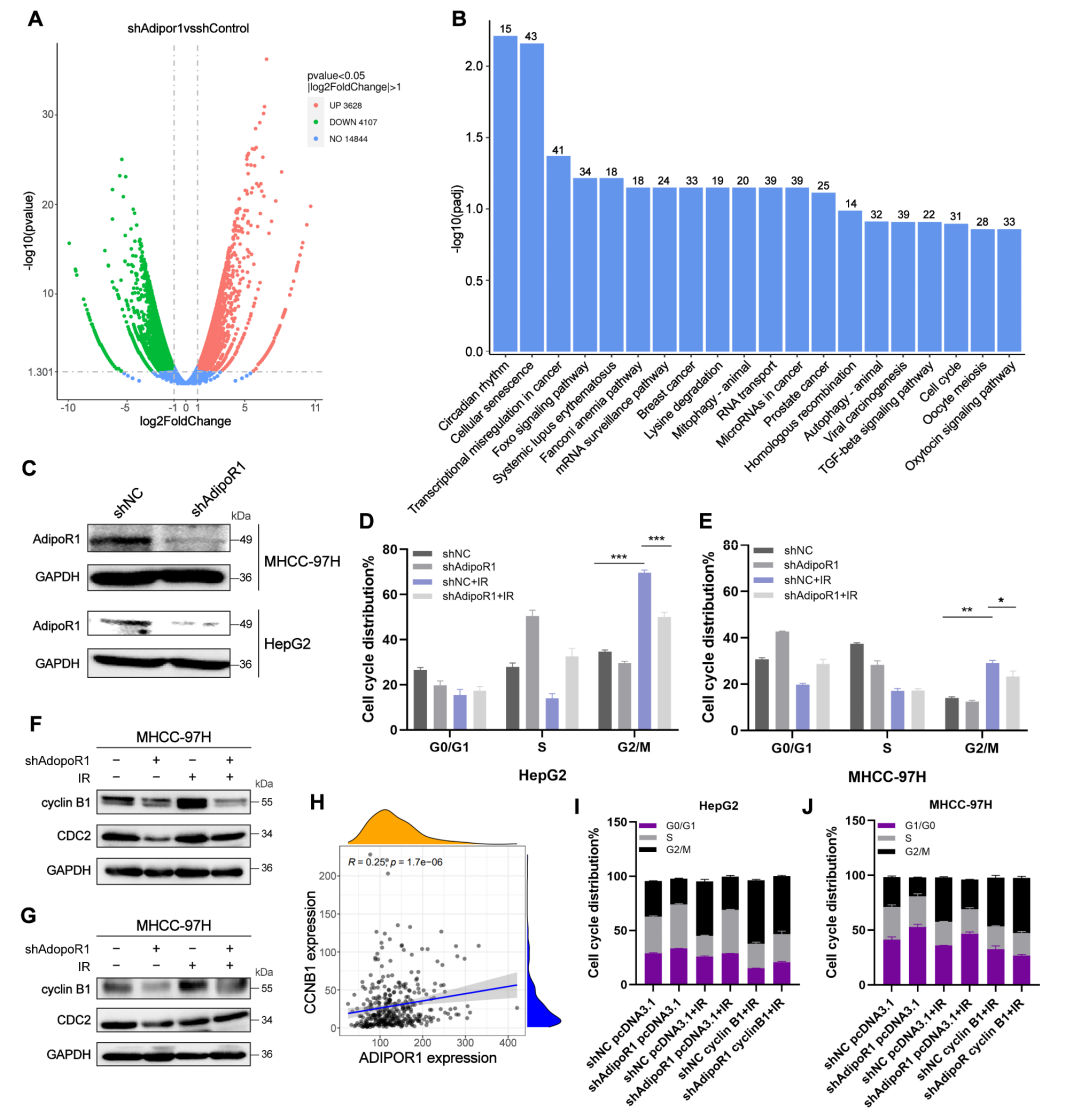
AdipoR1 regulates cell cycle through cyclin B1. (**A**)Volcano plot based on AdipoR1 transcriptome sequencing results; (**B**) Enrichment analysis of signaling pathways after AdipoR1 knockdown using RNA-seq data; (**C**)Western blot was used to detect the expression of AdipoR1 in MHCC-97 H and HepG2 cells after AdipoR1 knockdown; (**D-E**) MHCC-97 H and HepG2 cells after AdipoR1 knockdown were exposed to 10 Gy X-rays. The distribution of cells in each cycle was detected by PI staining and flow cytometry at 48 h after irradiation; (**F-G**) AdipoR1 knockdown MHCC-97 H and HepG2 cells were exposed to 10 Gy X-rays. The expression of cyclin B1 was detected by Western blot at 24 h after irradiation; (**H**)Scatterplot of correlation between AdipoR1 and cyclin B1 in hepatocellular carcinoma. The dark blue line is the linear regression line, and the gray area is the 95% confidence region; (**I-J**) cyclin B1 was overexpressed in AdipoR1 knockdown MHCC-97 H and HepG2 cells, and the distribution of the cell cycle was dertermined by flow cytometry after 24 h of ionizing radiation; P values were calculated using two-tailed unpaired student’s t-test. *p < 0.05, **p < 0.01, ***p < 0.001

To determine the role of AdipoR1 in liver cancer, a transcriptome of MHCC-97 H cells infected with AdipoR1 knockdown lentivirus was deeply sequenced. The volcano plot (Fig. 1A) displayed the up- and down-regulated genes as a result of AdipoR1 knockdown, and we applied KEGG (Kyoto Encyclopedia of Genes and Genomes) analysis to enrich the signaling pathways. The results suggested that the downstream genes after AdipoR1 knockdown were closely associated with the cell cycle pathway (Fig. 1B). To verify this, we knocked down AdipoR1 in MHCC-97 H and HepG2 cells. (Fig. 1C and S1A-B), as shown in (Fig. 1D-E), ionizing radiation could cause an arrest in the G2/M phase, while AdipoR1 knockdown could partially alleviate this effect, confirming the important role of AdipoR1 in cell-cycle progression. After detecting cell cycle-related key proteins, we found that the expression of cyclin B1 and CDC2 was down-regulated in AdipoR1 knockdown cells (Fig. 1F-G) and there is a positive correlation between AdipoR1 and cyclin B1 in hepatocellular carcinoma (Fig. 1H), suggesting that AdipoR1 may regulate cell cycle through cyclin B1. In order to further clarify the role of cyclin B1, we performed a rescue experiment by overexpressing CCNB 1 in the AdipoR1 knockdown model cells. As shown in (Fig. 1I-J), overexpression of cyclin B1 could reverse the alleviating effect of AdipoR1 knockdown on G2/M phase arrest. These findings suggest that the role o*f* AdipoR1 in IR-induced cell cycle arrest is closely related to cyclin B1.

### AdipoR1 regulates cyclin B1 through the ubiquitination-proteasomal degradation pathway

**Fig. 2:**
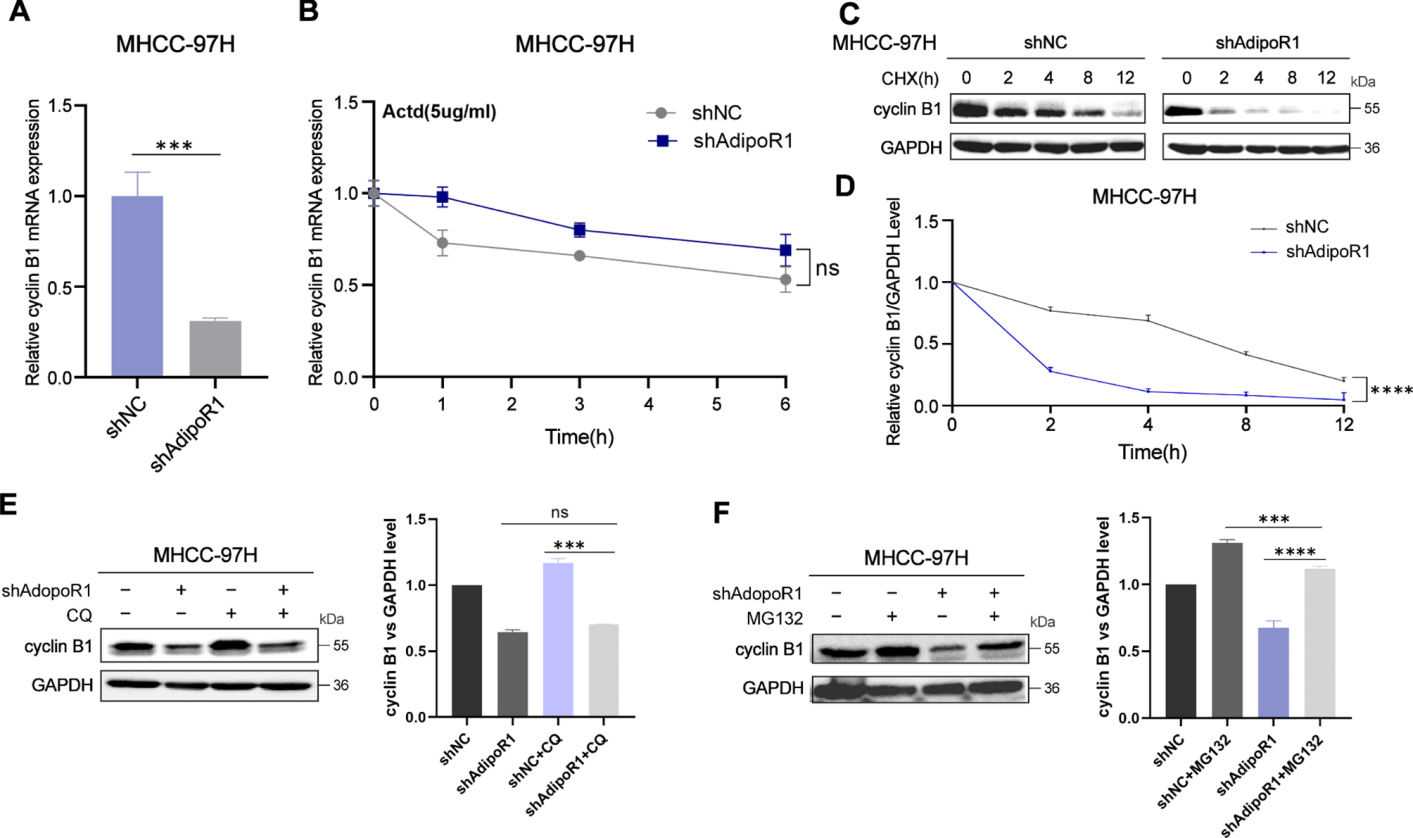
AdipoR1 regulates cyclin B1 through the ubiquitination-proteasomal degradation pathway. (**A**)The mRNA level of cyclin B1 in AdipoR1 knockdown MHCC-97 H cells was detected by QPCR; (**B**)MHCC-97 H cells with AdipoR1 knockdown were treated with the RNA synthesis inhibitor Actd (0, 1, 3, and 6 h). The mRNA level of cyclin B1 was detected by QPCR; (**C**)Protein stability of cyclin B1 in AdipoR1 knockdown MHCC-97 H cells was determined by western blotting after 0, 2, 4, 8 and 12 h of treatment with CHX (100 µg/ml); (**D**)The relative level of cyclin B1 protein was normalized with GAPDH, and the time course curve of CHX treatment was plotted; (**E**)Western blot detection of cyclin B1 expression in AdipoR1 knockdown MHCC-97 H cells treated with lysosomal inhibitor CQ (30 µM); (**F**)Western blot detection of cyclin B1 expression in AdipoR1 knockdown MHCC-97 H cells treated with proteasome inhibitor MG132 (10 µM); P values were calculated using two-tailed unpaired student’s t-test. ***p < 0.001, ****p < 0.0001

Next, we are going to explore the specific mechanisms by which AdipoR1 specifically regulates cyclin B1. Firstly, we found that AdipoR1 knockdown decreased the mRNA level of cyclinB1(Fig. 2A), while overexpression of AdipoR1 increased the mRNA level of cyclinB1(Fig. S1C), which suggested that AdipoR1 may affect cyclin B1 transcription. However, when we added Actinomycin D, an inhibitor of mRNA synthesis, to cells and administered it at various intervals, there was no significant difference compared to the shNC group (Fig. 2B). Therefore, we treated AdipoR1 knockdown cells with the protein synthesis inhibitor CHX, and the stability of the cyclin B1 protein was altered after 0, 2, 4, 8 and 12 h of treatment. In other words, AdipoR1 knockdown promoted the degradation of cyclin B1 (Fig. 2C–D). To determine the mechanism by which AdipoR1 degrades cyclin B1, AdipoR1 knockdown cells were treated with the proteasome inhibitor MG132 and the autophagy-lysosomal inhibitor CQ. As shown in (Fig. 2E-F), MG132 reversed the decrease in cyclin B1 expression in AdipoR1 knockdown cells, whereas CQ had no significant effect. Taken together, these results suggest that AdipoR1 regulates cyclin B1 through the ubiquitination degradation pathway. Interestingly, when we knocked down or overexpressed some common transcription factors to detect the mRNA level of cyclin B1, we found that FOXA1 and EGR1 may have some influence on cyclin B1 (Fig. S2A). It seems to imply that the mechanism by which we AdipoR1 regulate cyclin B1 is also not only limited to the protein level, which requires further exploration.

### AdipoR1 regulates cyclin B1 ubiquitination and degradation through the E3 ubiquitin ligase CCNB1IP1

**Fig. 3:**
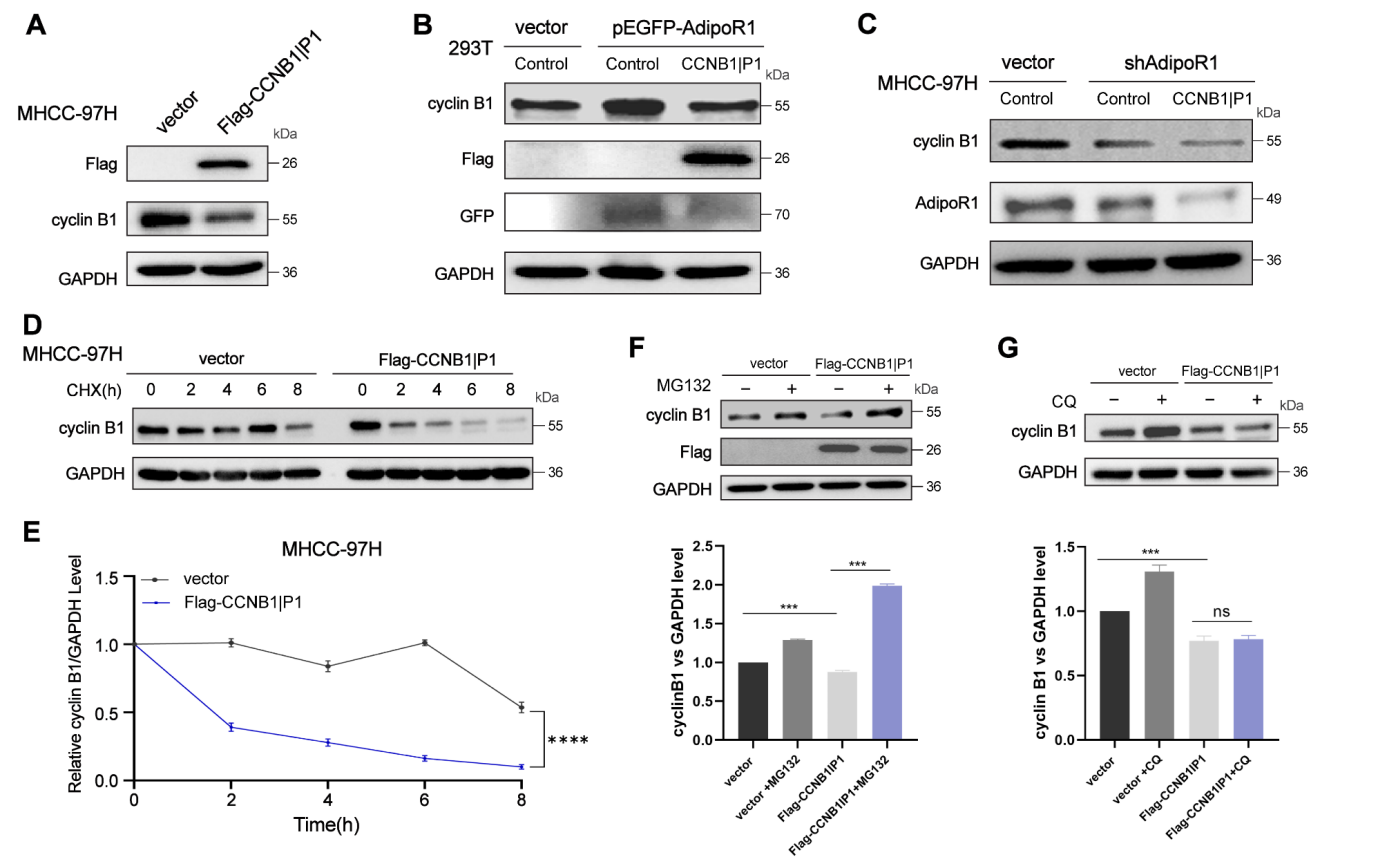
AdipoR1 regulates cyclin B1 ubiquitination and degradation through the E3 ubiquitin ligase CCNB1IP1. (**A**)Western blot was used to detect the expression of cyclin B1 in MHCC-97 H cells overexpressing CCNB1IP1; (**B**)CCNB1IP1 was transiently transfected into 293T cells overexpressing AdipoR1, and the expressions of cyclin B1, AdipoR1 and Flag proteins were analyzed by Western blot; (**C**)CCNB1IP1 was transiently transfected into MHCC-97 H cells with AdipoR1 knockdown, and the expressions of cyclin B1, AdipoR1 and Flag proteins were analyzed by Western blot; (**D**)MHCC-97 H cells overexpressing CCNB1IP1 were treated with cycloheximide (CHX) (100ug/ml), and cells were collected at 0, 2, 4, 8 and 12 h for Western blotting; (**E**)Relative levels of cyclin B1 protein were normalized with GAPDH, and the time course curve of CHX treatment was plotted; (**F**)MHCC-97 H cells overexpressing CCNB1IP1 were treated with MG132 (10 µM), and the cells were harvested 12 h later. The expression of cyclin B1 was analyzed by Western blot; (**G**)MHCC-97 H cells overexpressing CCNB1IP1 were treated with CQ (30 µM), and the cells were harvested 24 h later. The expression of cyclin B1 was analyzed by Western blot; *p < 0.05, **p < 0.01, ***p < 0.001 indicates statistical significance learning differences.P values were calculated using two-tailed unpaired student’s t-test

Based on the aforementioned findings, we wanted to further investigate the regulatory system in detail. First, GO analysis was performed using the transcriptome sequencing data. As shown in the bubble diagram (Fig. S3), AdipoR1 was supposed to regulate the ubiquitin protease activity. Since AdipoR1 is an adiponectin receptor, it has not been reported that it has ubiquitinase activity so far. We wonder if there is a ubiquitinase ligase between AdipoR1 and cyclin B1. We searched for the E3 ubiquitin ligase of cyclin B1 through the UbiBrowser database and intersected with the ubiquitinases that had changed in our transcriptomic sequencing results, finally, CCNB1IP1 was selected (Fig. S4). We constructed the CCNB1IP1 overexpression vector and transfected it into MHCC-97 H. As shown in (Fig. 3A), FLAG-CCNB1IP1 reduced the expression of cyclin B1. To determine whether CCNB1IP1 could function in the AdipoR1-mediated downregulation of cyclin B1, we simultaneously overexpressed CCNB1IP1 in AdipoR1 overexpression cells, and western blot results showed that AdipoR1 overexpression increased cyclin B1 levels, while overexpression of CCNB1IP1 could prevent the increase (Fig. 3B). In contrast, as shown in (Fig. 3C), overexpression of CCNB1IP1 in AdipoR1-knockdown cells accelerated the degradation of cyclin B1. Additionally, CHX treatment significantly decreased the protein level of cyclin B1 in CCNB1IP1 overexpression cells (Fig. 3D-E). Furthermore, we found that the expression of cyclin B1 in CCNB1IP1 overexpression cells was accumulated by MG132 but not CQ (Fig. 3F-G**)**. Taken together, these findings suggest that AdipoR1 reduces cyclin B1 expression through the E3 ubiquitin ligase CCNB1IP1 in hepatoma cells.

### CCNB1IP1 promotes the ubiquitination degradation of cyclin B1

**Fig. 4:**
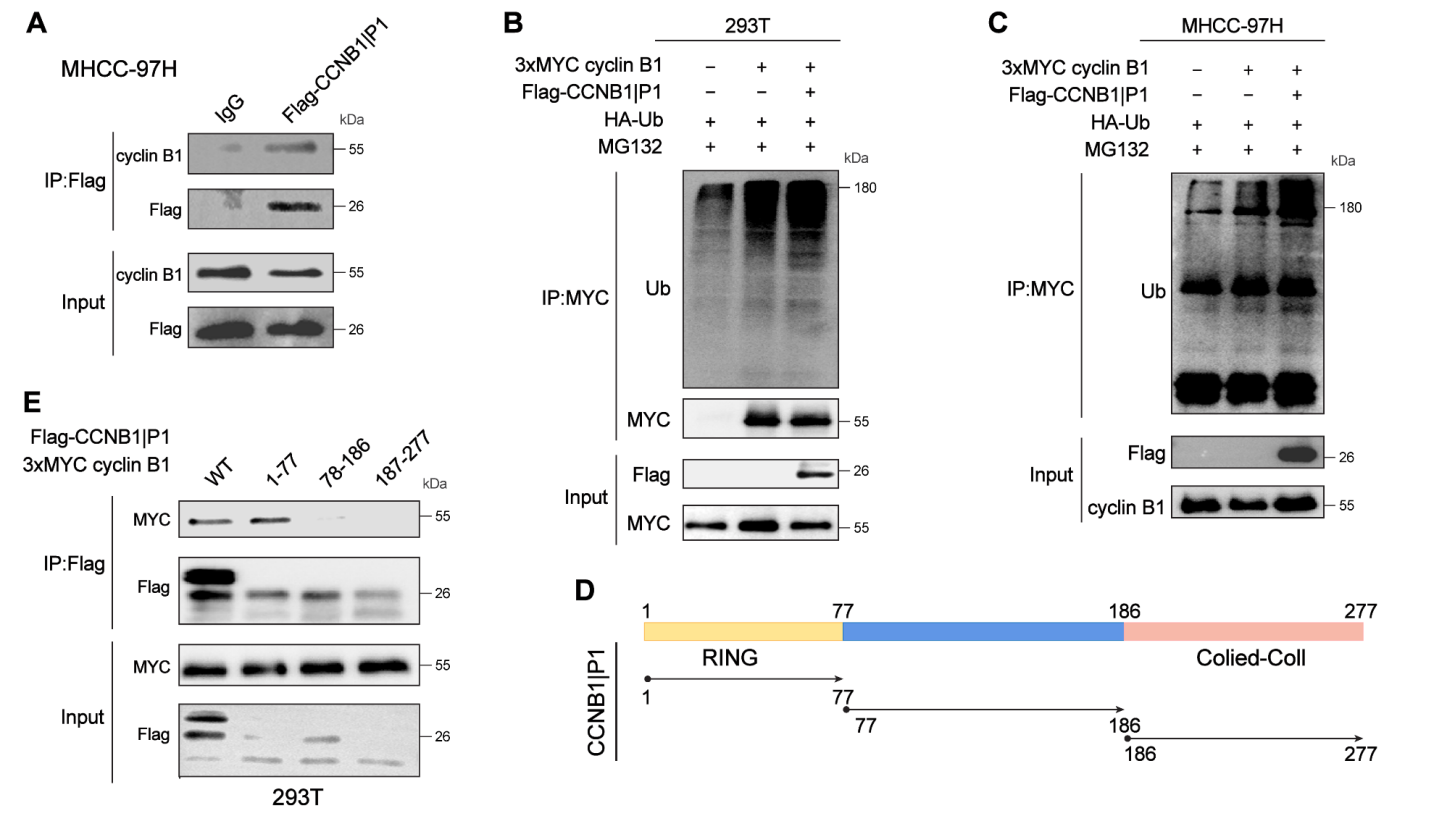
CCNB1IP1 promotes ubiquitination degradation of cyclin B1 and may play a role in the 1–77 domain. (**A**)Immunoprecipitation detection of MHCC-97 H cells transfected with FLAG-tagged CCNB1IP1 plasmid; (**B-C**)Flag-CCNB1IP1, 3xMyc-cyclin B1, and HA-Ub plasmid were co-transfected into 293T and MHCC-97 H cells. The ubiquitination of cyclin B1 was detected by immunoprecipitation; (**D**)Schematic diagram of CCNB1IP1 domain truncation; (**E**)The interaction between different domains of CCNB1IP1 and cyclin B1 was detected by immunoprecipitation

To confirm the interaction between CCNB1IP1 and cyclin B1, we transfected the FLAG-tagged CCNB1IP1 vector in MHCC-97 H cells and performed co-immunoprecipitation (Co-IP) studies. As expected, FLAG-tagged CCNB1IP1 could immunoprecipitate cyclin B1(Fig. 4A). And as shown in (Fig. 4B-C), CCNB1IP1 significantly increased cyclin B1 ubiquitination. To further clarify the interaction domain between CCNB1IP1 and cyclin B1, we used two protein structure analysis websites (NCBI: http://www.ncbi.nlm.nih.gov/, UniProt: https://www.UniProt.org/) to divide CCNB1IP1 into three domains, corresponding to the amino acid sequences at 1–77, 78–186 and 187–277 of the full sequence of CCNB1IP1 (Fig. 4D). As shown in (Fig. 4E), only the channels of WT and 1–77 truncates developed bands of MYC, indicating that CCNB1IP1 interacts with the 1–77 domain of cyclin B1.

### AdipoR1 regulates CCNB1IP1 transcription and translation through the transcriptional repressor ESR1

**Fig. 5:**
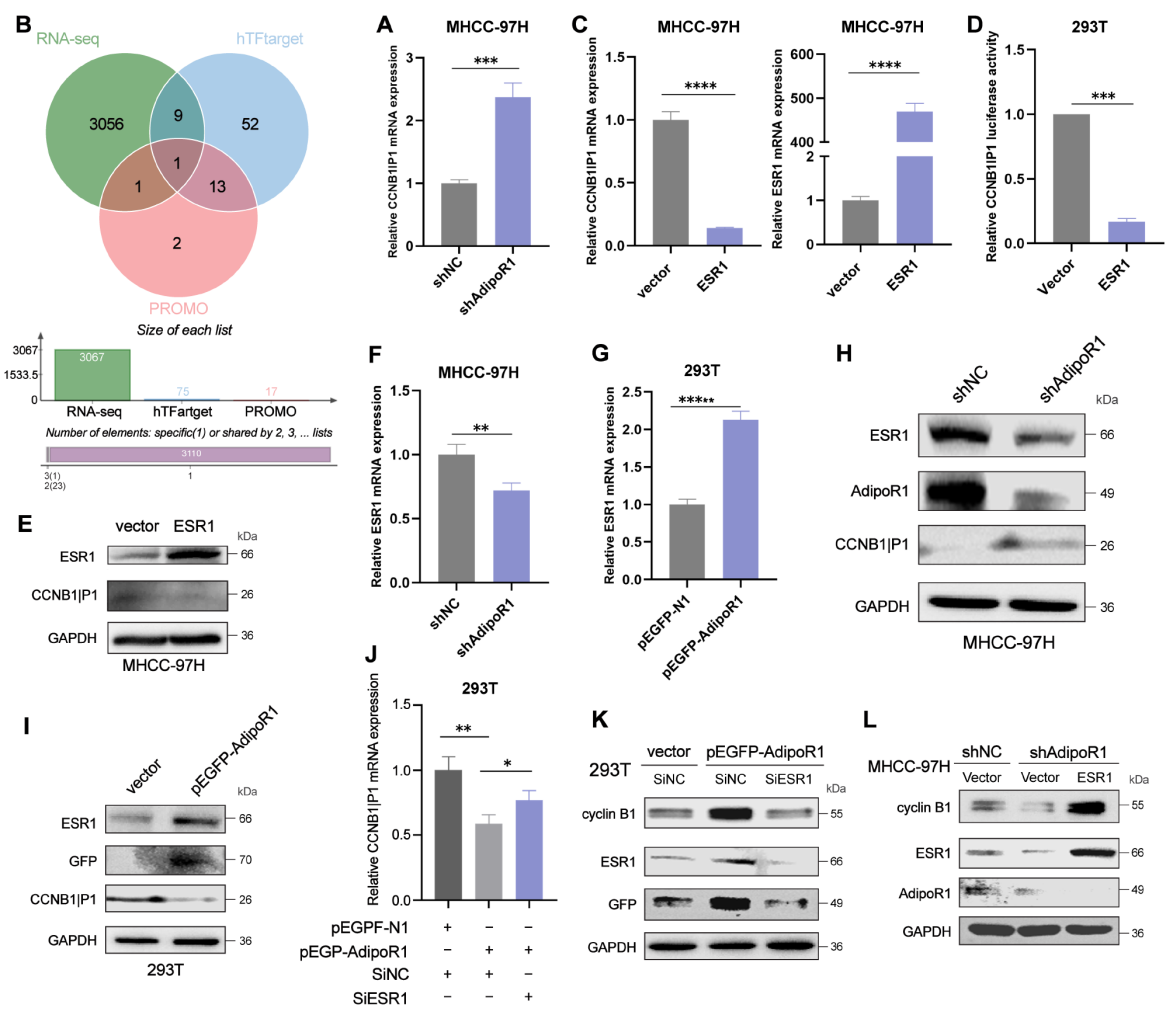
AdipoR1 regulates CCNB1IP1 transcription and translation through the transcriptional repressor ESR1. (**A**)QPCR was used to detect the mRNA level of CCNB1IP1 in MHCC-97 H AdipoR1 knockdown cells; (**B)**Venn diagram demonstrates that genes in the two transcription factor databases, HIF Target and Promo, overlap with the genes of AdipoR1 down-regulated genes; (**C**)QPCR was used to detect the mRNA level of CCNB1IP1 in ESR1-overexpressing MHCC-97 H cells; (**D**)Dual-luciferase reporter gene assay was used to detect the promoter activity of CCNB1IP1 in ESR1- overexpressing MHCC-97 H cells; (**E**)Western blot was used to detect the expression of CCNB1IP1 in ESR1- overexpressing MHCC-97 H cells; (**F-G**)QPCR was used to detect the expression of ESR1 after AdipoR1 knockdown or overexpression; (**H-I)**Western blot was used to detect the expression of ESR1 after AdipoR1 knockdown or overexpression; (**J**) siESR1 was transiently transfected into AdipoR1-overexpressing 293T cells, and the mRNA level of CCNB1IP1 was detected by QPCR; (**K**)Si-ESR1 was transiently transfected into AdipoR1-overexpressing 293T cells, and the expression of AdipoR1, ESR1 and cyclin B1 was detected by Western blot; (**L**)ESR1 was overexpressed in MHCC-97 H cells with AdipoR1 knockdown. The expressions of AdipoR1, ESR1 and cyclin B1 were detected by Western blot; P values were calculated using two-tailed unpaired student’s t-test. *p < 0.05, **p < 0.01, ***p < 0.001, ****p < 0.0001

Given that the underlying molecular mechanism by which AdipoR1 could control CCNB1IP1 remains unclear. We first detected the mRNA expression of CCNB1IP1 after AdipoR1 knockdown, as shown in (Fig. 5A), AdipoR1 knockdown significantly increased the mRNA level of CCNB1IP1. Next, we further explored the transcriptional mechanism of CCNB1IP1 promotion by AdipoR1. Through the enrichment analysis of the down-regulation results of AdipoR1 sequencing genes and the two transcription factor databases of HIF Target and Promo, as shown in the Venn diagram (Fig. 5B), ESR1 is the only transcription factor that intersected all three of the databases. To test this hypothesis, we transfected MHCC-97 H with siRNAs or overexpression plasmids of serveral transcription factors. As shown in (Fig. S2B), these transcription factors had no significant effect on the mRNA level of CCNB1IP1, regardless of silencing or overexpression. As expected, only the transcription factor ESR1 had an impact on the mRNA level of CCNB1IP1, indicating that overexpressing ESR1 suppressed CCNB1IP1 expression (Fig. 5C). Dual-luciferase reporter gene experiments also showed that overexpression of ESR1 reduced CCNB1IP1 promoter activity (Fig. 5D). Thus, ESR1 acted as a transcriptional repressor to negatively regulate CCNB1IP1. Notably, overexpression of ESR1 can also alter the protein expression of CCNB1IP1 (Fig. 5E). Next, in order to clarify the relationship between AdipoR1 and ESR1, we detected changes in ESR1 when AdipoR1 was overexpressed or knocked down from the mRNA and protein levels, respectively. As shown in Fig. 5F-I, AdipoR1 could positively regulate ESR1 at the mRNA and protein levels. To explore whether AdipoR1 affected CCNB1IP1 through the transcriptional repressor ESR1 and thereby regulated cyclin B1, we transiently transfected AdipoR1 overexpression cells with ESR1-siRNA to detect the mRNA level of CCNB1IP1 and the protein expression of cyclin B1, respectively. As shown in (Fig. 5J), although the mRNA level of CCNB1IP1 decreased after AdipoR1 overexpression, ESR1-siRNA reversed the downregulation of CCNB1IP1 after AdipoR1 overexpression. Likewise, AdipoR1 promoted the expression of cyclin B1, but ESR1-siRNA reversed the upregulation of cyclin B1 by AdipoR1 overexpression (Fig. 5K). We reversed ESR1 in AdipoR1 knockdown cells, and as shown (Fig. 5L), overexpression of ESR1 significantly reversed the downregulation of cyclin B1. Based on the above findings, we wanted to explore the relationship between AdipoR1 and ESR1. Interestingly, we found that AdipoR1 did not function in a protein-bound manner with ESR1 (Fig. S5A). Since the nucleus is where transcription factors control gene transcription, we speculated that AdipoR1 is able to promote the entry of the transcription factor ESR1 into the nucleus. As expected, overexpression of AdipoR1 could promote the expression of ESR1, and the protein was more distributed in the nucleus (Fig. S5B). In conclusion, AdipoR1 can regulate CCNB1IP1 by promoting increased nuclear localization of transcription factor ESR1 and then regulating cyclin B1.

### Cyclin B1 expression reverses the effect of AdipoR1 knockdown on radiosensitivity in HCC cells

**Fig. 6:**
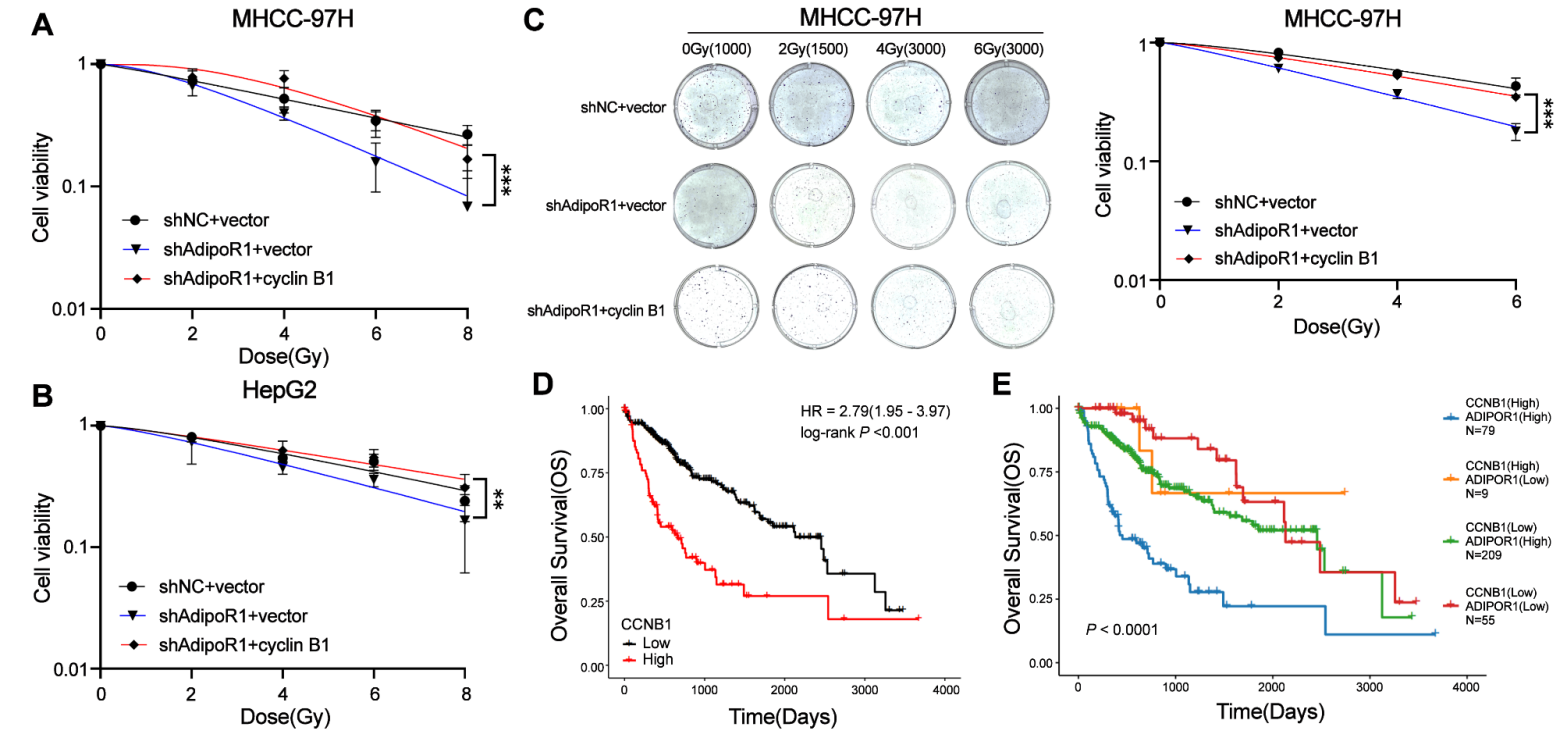
Cyclin B1 expression reverses the effect of AdipoR1 knockdown on radiosensitivity in HCC cells. (**A-B**).Cyclin B1 was overexpressed in AdipoR1 knockdown MHCC-97 H and HepG2 cells, and cell viability under different doses of X-ray irradiation was detected by CCK8 assay; (**C**) Evaluation of MHCC-97 H cell proliferation using colony formation assays. Error bars show the mean ± SD. ***, *p* < 0.001; (**D**) Kaplan-Meier survival of cyclin B1 expression levels in TCGA database curves; (**E**)Kaplan-Meier survival curves of high and low expression levels of cyclin B1 and AdipoR1 in TCGA database

Studies have shown that G2/M cell cycle arrest is negatively correlated with radiation sensitivity. In previous studies, we confirmed that AdipoR1 knockdown can inhibit G2/M phase arrest induced by ionizing radiation. Therefore, we speculated whether AdipoR1 knockdown would lead to increased radiosensitivity. As shown **(**Fig. 6A-C), AdipoR1 knockdown increased the radiosensitivity of HCC cells, while overexpression of cyclin B1 reversed the effect of knockdown of AdipoR1 on the radiosensitivity of HCC cells. We also used R software to analyze TCGA-LIHC data, discovering that high expression of cyclin B1 was closely related to poor the prognosis of HCC patients (Fig. 6D). Besides, the combined group of high cyclin B1 expression and high AdipoR1 expression showed the worst prognosis (Fig. 6E). Taken together, these findings further confirmed that AdipoR1-mediated radiosensitivity of HCC cells and the prognosis of HCC patients were at least partially influenced by cyclin B1.

## Discussion

Protein post-translational modifications play key roles in many biological processes by regulating protein functions. To date, more than 450 unique protein modifications have been identified, including phosphorylation, acetylation, ubiquitination, and sumoylation. These modifications can alter target protein activity, intracellular distribution, protein interactions, and protein longevity (Li et al. 2021; Huang et al. 2018; Chen et al. 2020). Ubiquitination is one of the types of post-translational modifications that plays a crucial role in controlling the degradation of substrates. Dysregulation of ubiquitination leads to uncontrolled cell cycle progression and ultimately tumorigenesis; therefore, targeting the ubiquitin system may provide an effective therapeutic strategy for cancer therapy (Dang et al. 2021). Ubiquitination is an ATP-dependent three-step enzymatic cascade that requires the participation of Ub-activating enzyme E1, Ub-conjugating enzyme E2, and Ub ligase E3 (Cai et al. 2018). Among them, E3 ligase is the most important component in the Ub coupling mechanism, and its mediated degradation has high substrate specificity (Deng et al. 2020). CCNB1IP1, also known as human invasion enhancer 10 (HEI10), mediates meiotic recombination and plays a role in cell proliferation (Muyt et al. 2014). Identified from a HeLa cell library as early as 2003 and first identified as a RING finger family ubiquitin ligase that regulates the cell cycle by interacting with cyclin B1 and promoting its degradation, thereby playing a role in progression through the G2/M effect (Toby et al. 2003). In the present study, our results have suggested that CCNB1IP1 is an E3 ubiquitin ligase upstream of cyclin B1, and AdipoR1 could regulate cyclin B1 degradation through CCNB1IP1. We also found that the 1–77 domain of CCNB1IP1 had the strongest binding ability to cyclin B1, which may be related to the zinc finger domain contained in 1–77. Our results further demonstrated the ability of CCNB1IP1 to ubiquitinate cyclin B1, thus targeting the E3 ubiquitin ligase involved in cell cycle regulation is expected to provide new therapeutic strategies for cancer therapy.

Transcription factors are a group of proteins with DNA-binding domains that can bind to DNA strands of promoters or enhancers and initiate gene transcription under the coordinated action of RNA polymerase and other cofactors, which are important for embryogenesis and development. At the same time, transcription factors also play a focal role in various signaling pathways, controlling cell growth, proliferation, metabolism, apoptosis, and other processes (Chen and Koehler 2020; Li et al. 2022a, b). Estrogen receptor 1 (ESR1) is a ligand-induced steroid hormone receptor that mediates estrogen signaling and regulates transcriptionally driving growth, proliferation, and differentiation including cellular processes, in many breast and uterine It functions as an oncogene in endometrial tumors (Blanchard et al. 2019; Shanle and Xu 2010). Since ESR1 is a member of the nuclear receptor superfamily and can translocate into the nucleus upon activation by estrogen, it may also function as a transcription factor (Nilsson and Gustafsson 2011). In this study, we first screened out the transcription factor ESR1 through enrichment analyses. As we expected, ESR1 could inhibit the mRNA level of CCNB1IP1. Dual-luciferase reporter gene assay results showed that ESR1 could inhibit the promoter activity of CCNB1IP1, and our results also confirmed that ESR1 was a transcriptional repressor of AdipoR1, regulating CCNB1IP1 transcription. Next, we wanted to explore how AdipoR1 regulated ESR1. We found that AdipoR1 could not function in a protein-binding manner with ESR1, which led us to further thinking. It is well known that transcription factors exert their transcriptional role in the nucleus. We wondered whether AdipoR1 could also promote the entry of ESR1 into the nucleus. Later, we observed that the nuclear protein distribution was higher in AdipoR1 overexpression cells, and immunofluorescence experiments also confirmed that AdipoR1 could increase the nuclear localization of ESR1.

Adiponectin (APN) is an adipocyte-differentiated cytokine that plays a key role in regulating many physiological processes by binding to specific receptors (Zhang et al. 2022). There are two main forms of adiponectin receptors: adiponectin receptor 1 (AdipoR1) and adiponectin receptor 2 (AdipoR2). AdipoR1 and AdipoR2 have been identified in various tissues and cell types, and their expression has been reported in human lung, breast, pancreatic, colorectal, and gastric cancer tissues (Byeon et al. 2010). Our previous findings highlighted the role of AdipoR1 in liver cancer, proving that AdipoR1 was highly expressed in liver cancer and that knockdown of AdipoR1 could promote cell death, which is also consistent with the results of this study. In this study, we found that knockdown of AdipoR1 promoted the occurrence of mitotic catastrophe; however, the specific mechanism is not yet clear, and further research is needed. AdipoR1 has been reported to function by activating the AMPK pathway. Under high glucose conditions, adiponectin can bind to cell membrane surface receptors, activate the AMPK pathway, and significantly increase insulin secretion, thereby reducing blood sugar (Xiaohu et al. 2022). Recent studies have shown that MiR-221 can mediate the epithelial-mesenchymal transition of hepatocellular carcinoma by targeting AdipoR1, a discovery that will improve the understanding of the mechanism of EMT progression and provide a new target for molecular therapy of hepatocellular carcinoma point (Li et al. 2017a, b). AdipoRon, a small-molecule agonist of the APN receptor, selectively activates AdipoR1 and AdipoR2, which are reported to readily penetrate the blood-brain barrier and exert the same effects as APN in the central nervous system. However, the role of AdipoRon in tumor cells has not yet been known, which is worthy of further exploration (Zhang et al. 2022).

Radiotherapy combined with radiosensitizers is an attractive strategy to improve treatment rates for HCC patients. In selected HCC patients, radiotherapy combined with chemotherapy has shown good tumor response and extended survival (Lee and Seong 2011). Oxaliplatin is one of the most commonly used chemotherapy drugs for HCC and has been widely studied for patients with advanced liver cancer. Even so, oxaliplatin resistance is also an important cause of poor therapeutic effect and relapse in HCC (Sun et al. 2021; Abdel-Rahman 2014). In this study, we treated MHCC-97 H and HepG2 cells with AdipoR1 knockdown, oxaliplatin, IR or combined treatment, respectively. The results showed that before ionizing radiation, compared with the oxaliplatin alone treatment group, AdipoR1 knockdown combined with oxaliplatin treatment reduced cell viability. AdipoR1 knockdown combined with oxaliplatin can significantly promote radiotherapy efficacy (Fig. S6A-B). However, this conclusion needs to be confirmed in clinical samples and animal studies. In this result, we also found that AdipoR1 knockdown could inhibit the proliferation of hepatocellular carcinoma cells before and after irradiation, and this inhibitory effect was more obvious in the AdipoR1 knockdown combined irradiation group. This result is consistent with the conclusion obtained in our previous rat experiments. Compared with the shNC group, the tumor volume of the shAdipoR1 group decreased after treatment, while that of sh-Adipor1 + IR group was smaller than that of the IR group (Liu et al. 2022).

Different from previous studies, for the first time our data demonstrated the relationship between AdipoR1, cell cycle arrest, and radiation sensitivity and found that ionizing radiation regulated cell cycle and radiation sensitivity through the AdipoR1/ESR1/CCNB1IP1/CCNB1 signaling pathway. These results suggested a close link between the AdipoR1-regulated cell cycle and radiosensitivity in HCC, providing new ideas for the study of targeted radiotherapy.

## Conclusion

**Fig. 7:**
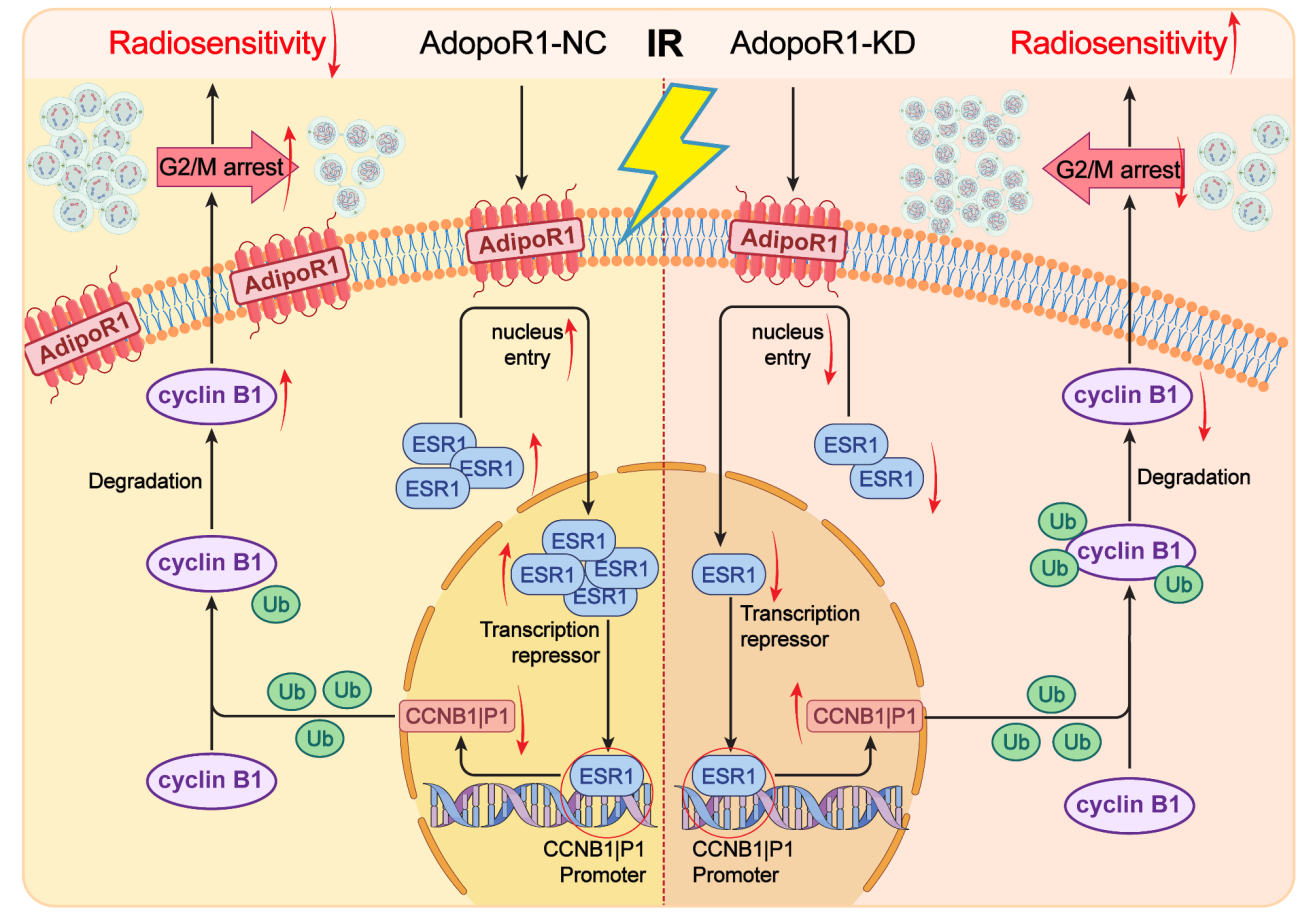
Schematic diagram showing the role of AdipoR1 in ionizing radiation-induced cell cycle arrest and radiation sensitivity

Taken together, we found a new role for AdipoR1 in cell cycle and ubiquitination regulatory mechanisms. AdipoR1 regulates cyclin B1’s ubiquitin ligase CCNB1IP1 through the transcriptional repressor ESR1, causing changes in cell cycle distribution and thereby affecting the radiosensitivity of HCC cells. We conclude that the"AdipoR1-ESR1-CCNB1IP1-cyclin B1” axis has potential implications for developing predictive biomarkers and therapeutic targets in HCC patients (Fig. 7).

## Electronic supplementary material

Below is the link to the electronic supplementary material.

Supplementary Material 1

Supplementary Material 2

## Data Availability

No datasets were generated or analysed during the current study.

## Code availability

R package tidyverse, jsonlite and R.utils were used for data processing of liver cancer expression profile downloaded by GDC. R package survminer and survival are used for KM survival analysis. R packages ggpubr and ggExtra were used for correlation analysis between ADIPOR1 and CCNB1. Use of R-package clusterProfiler for gene enrichment analysis.
