# Supplementary material for: AdipoR1 enhances the radiation resistance via ESR1/CCNB1IP1/cyclin B1 pathway in hepatocellular carcinoma cells
Source: Mol Med. 2025 Jan 23;31:21. doi: 10.1186/s10020-025-01065-0 (PMC11755959; doi:10.1186/s10020-025-01065-0)
Supplement: Supplementary file 1 — Supplementary Material 1 [file 10020_2025_1065_MOESM1_ESM.docx]

**Supplementary appendix**

**
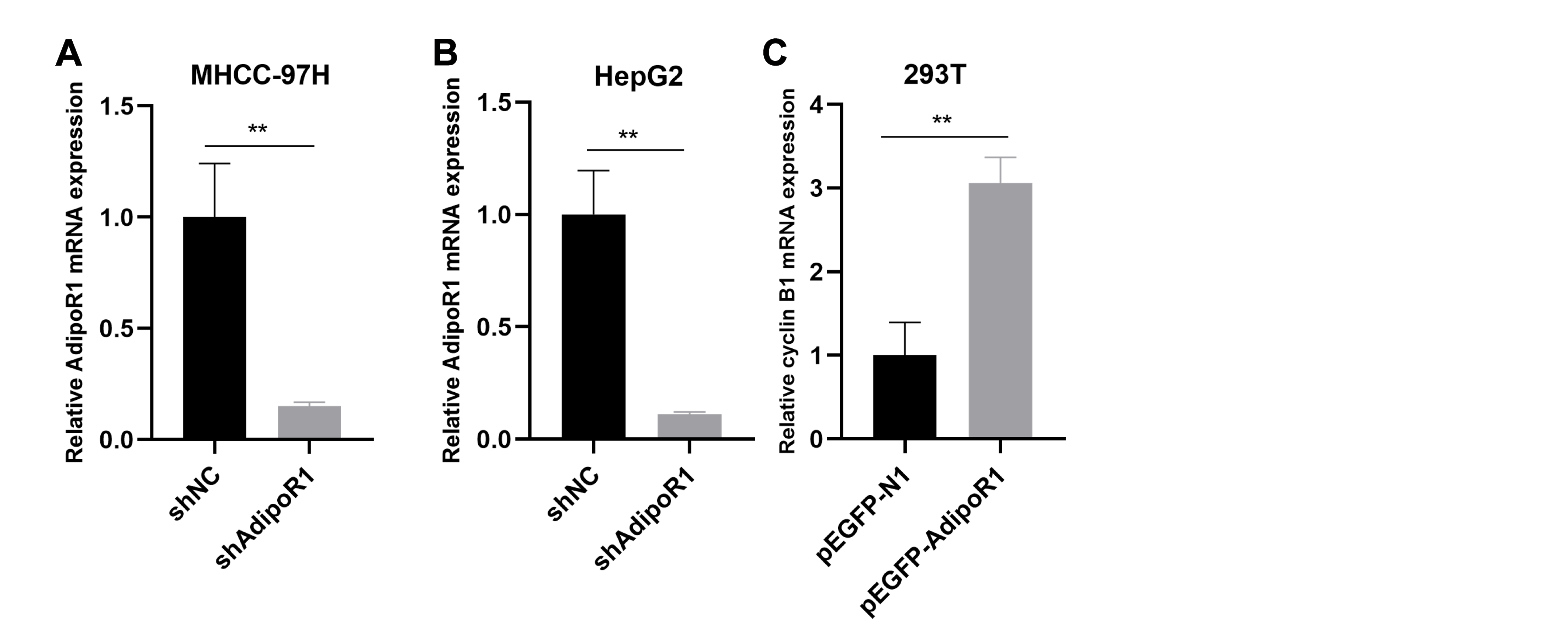
**

**Supplementary Fig 1. Knockdown effect of AdipoR1 on mRNA level and effect of overexpression of AdipoR1 on cyclin B1 mRNA level.**

(A-B)**:** QPCR was used to detect the knockdown effect of AdipoR1 in MHCC-97H and HepG2 cells; (C): The mRNA level of cyclin B1 after AdipoR1 overexpression was detected by QPCR. **P* < 0.05, ***P* < 0.01, ****P* < 0.001 indicates a statistical difference.

**
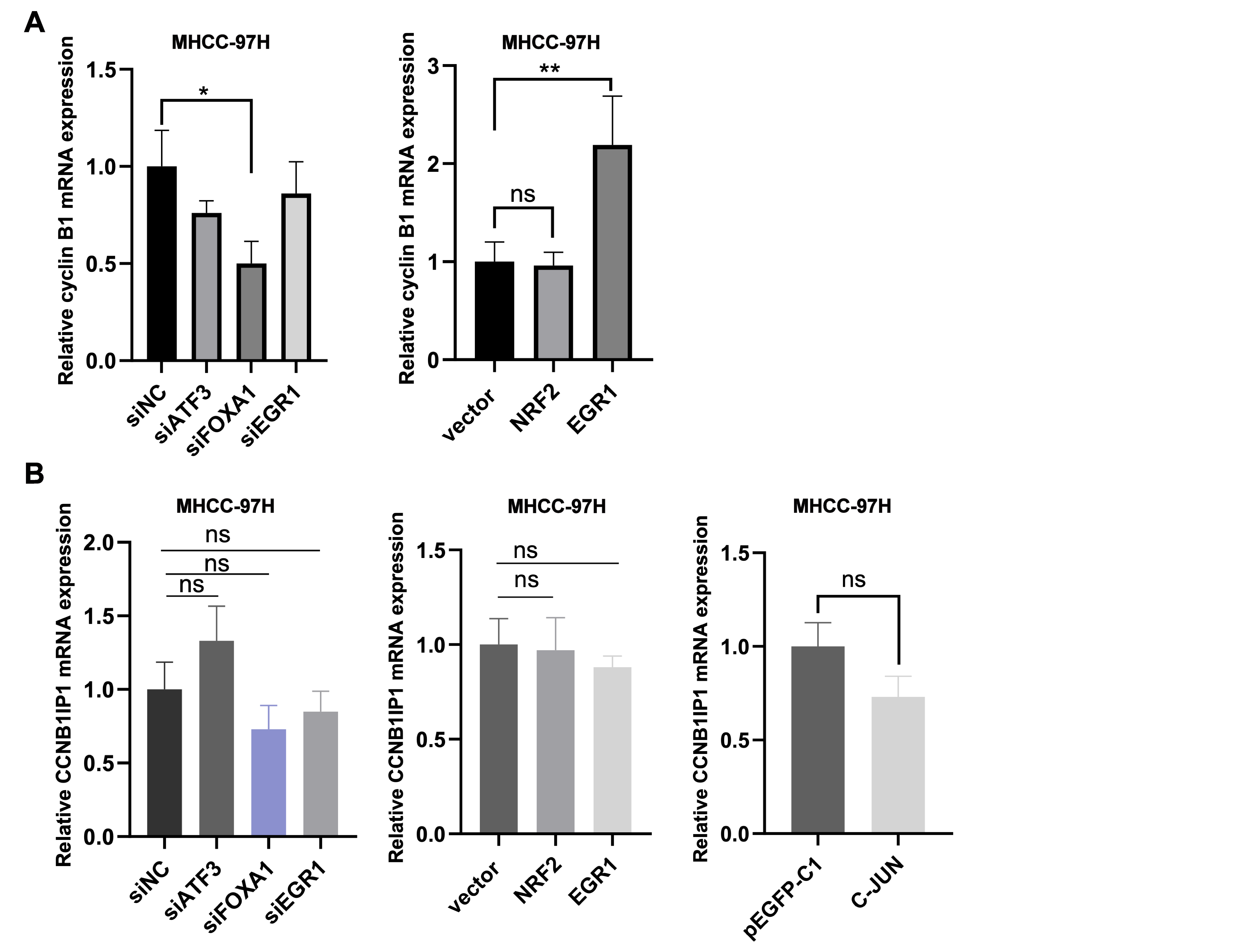
**

**Supplementary Fig 2. Effects of some transcription factors on mRNA levels of cyclin B1 and CCNB1IP1.**

(A): QPCR was used to detect the effect of transient transfection of siATF3, siFOXA1 and siEGR1 on the mRNA level of cyclin B1 in MHCC-97H cells; QPCR was used to detect the effect of NRF2 and EGR1 overexpression on the mRNA level of cyclin B1 in MHCC-97H cells; (B): QPCR was used to detect the effect of transient transfection of siATF3, siFOXA1 and siEGR1 on the mRNA level of CCNB1IP1 in MHCC-97H cells; QPCR was used to detect the effect of NRF2, EGR1 and C-Jun overexpression on the mRNA level of CCNB1IP1 in MHCC-97H cells.

**
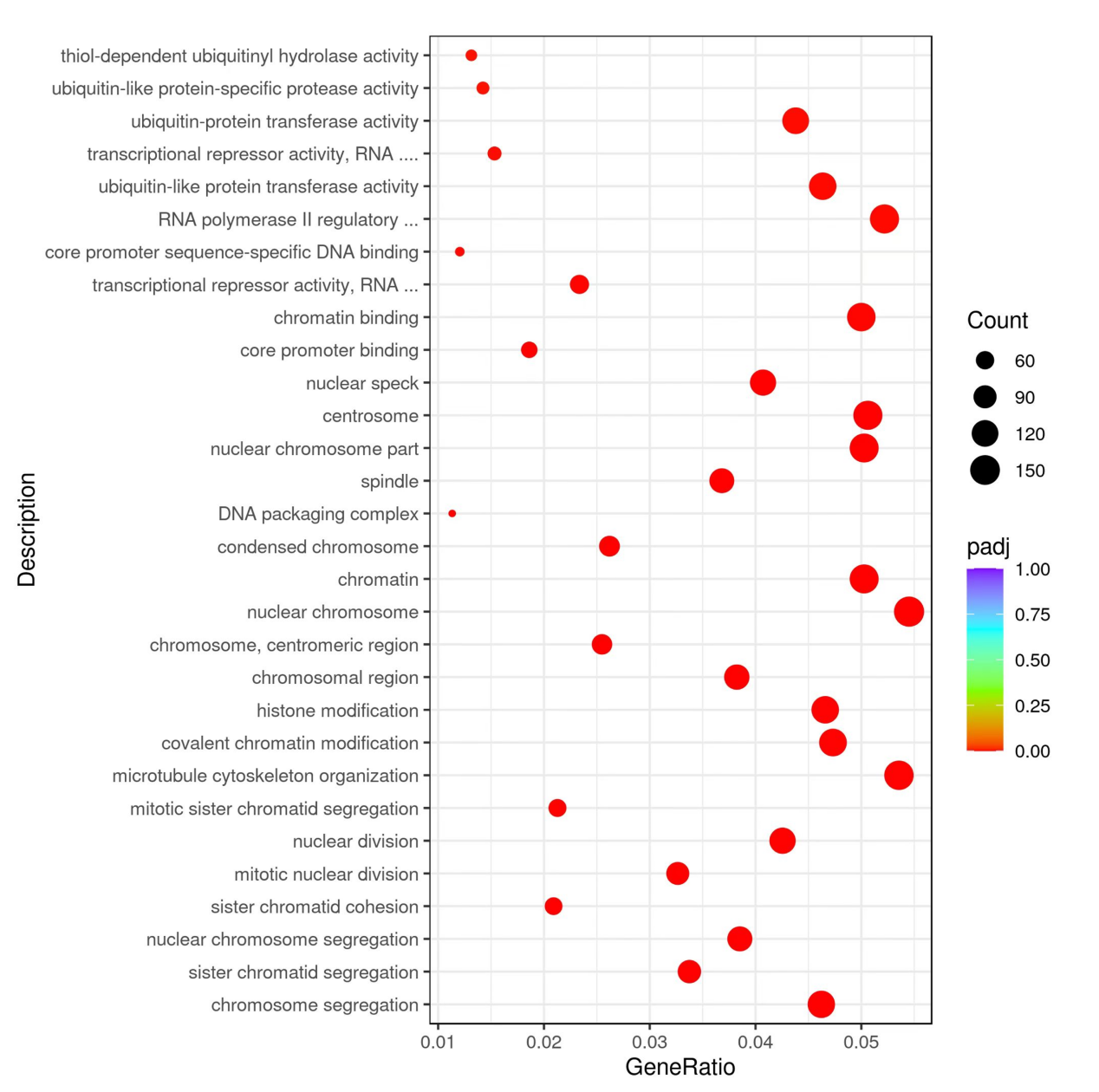
**

**Supplementary Fig 3. GO analysis bubble diagram based on transcriptome sequencing results of AdipoR1 knockdown group and control group.**


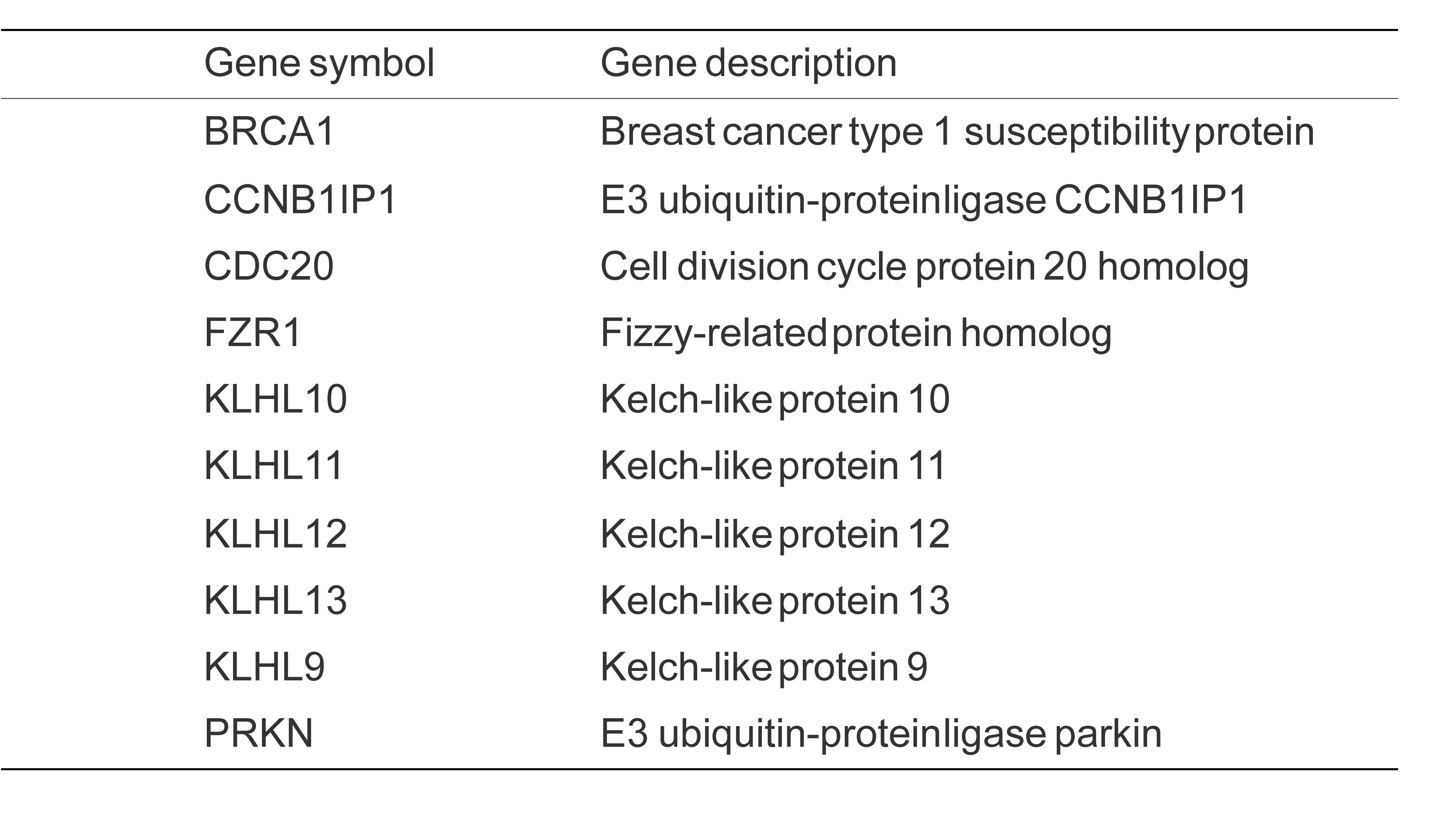


**Supplementary Fig 4. The three-line table shows the E3 ubiquitin ligases predicted using cyclin B1 as substrate by UbiBrowser Database.**

**
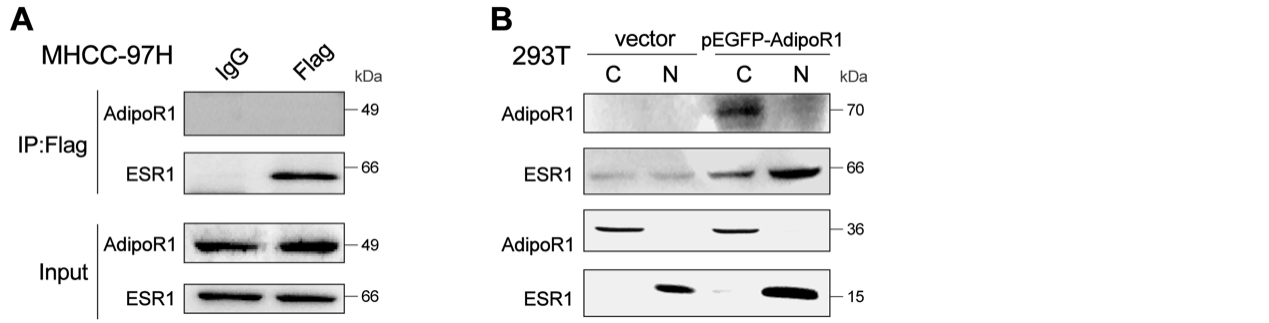
**

**Supplementary Fig 5. AdipoR1 can promote increased nuclear localization of transcription factor ESR1.**

(**A**): Immunoprecipitation detection of MHCC-97H cells transfected with FLAG-tagged ESR1 plasmid; (**B**): The subcellular distribution of ESR1 in vector-transfected and AdipoR1-overexpressing 293T cells was analyzed by Western blot.


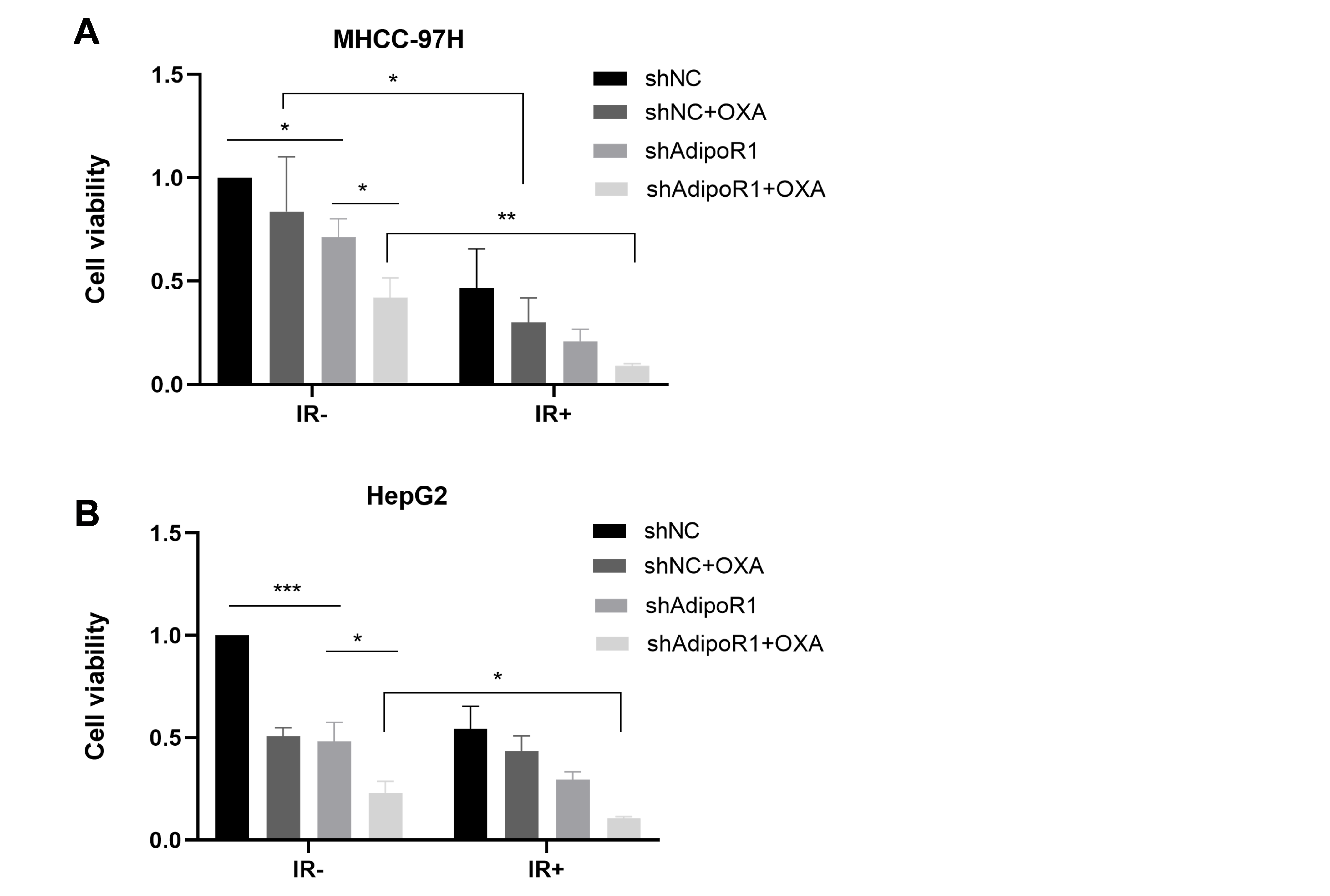


**Supplementary Fig 6. AdipoR1 knockdown combined with oxaliplatin promotes radiotherapy efficacy.**

(A-B): AdipoR1 knockdown, oxaliplatin, IR, or combination treatment were used in MHCC-97H and HepG cells, and then cell viability was measured by CCK8 assay.The concentration of oxaliplatin is 10uM. **P* < 0.05, ***P* < 0.01, ****P* < 0.001 indicates a statistical difference.
